# Supplementary material for: Implementation of a Vaccination Program Based on Epidemic Geospatial Attributes: COVID-19 Pandemic in Ohio as a Case Study and Proof of Concept
Source: Vaccines (Basel). 2021 Oct 25;9(11):1242. doi: 10.3390/vaccines9111242 (PMC8625927; doi:10.3390/vaccines9111242)

SUPPLENENTARY MATERIALS FOR

Implementation of a vaccination program based on epidemic geospatial attributes: COVID-19 pandemic in Ohio as a case study and proof of concept

MODEL DESCRIPTION

We developed a spatial deterministic compartmental mathematical model to simulate the impact of vaccine and dynamics of confirmed COVID-19 cases, COVID-19-related hospitalizations and ICU admissions, and COVID-19-related deaths in the state of Ohio. The model incorporated spatial connectivity information by county. The COVID-19 spatial was formulated as a system of coupled nonlinear differential equations that stratify the population into compartments according to spatial risk group, infection status, hospitalization and disease stage, and vaccination state.

The model stratified the population into four different spatial groups depending of spatial risk characteristics of the county. The spatial risk groups were defined as following, *Group 1*: counties with airports; *Group 2*: counties surrounding the counties with airports; *Group 3*: counties with main highways crossing the county; and *Group 4*: counties not surrounding counties with airports or being crossed by main highways. Each group has its own dynamic of the disease and the directional connections between groups are assumed to be by flow of infections between the different groups at specific rates. The dynamic of the disease transmission and infection complications in absence or presence of vaccine in each group was modeled using seven epidemiological compartments for the susceptible, infected, recovered, hospitalized, ICU admitted, recovered after hospitalization, and death population. Susceptible individuals in each spatial group are at risk of being exposed to infection at varying hazard rates, which are group- and time dependent, to capture the variability in the risk of exposure and the impact of public health interventions.

Susceptible individuals in *Group i* (*i*= 1, 2, 3, 4) get infected at a group specific hazard rate λ_i. Infected individuals can recover at a rate δ, or can get hospitalized at rate η_i. Infected individuals can die of COVID-19 infection complications without get hospitalized at rate ψ. Hospitalized individuals can recover at rate σ or an get admitted to ICU at rate ω_i. COVID-19 infection complications admitted to ICU can recover at a rate ξ, or patients die at a rate μ_i. Spatial risk groups are connected by a flow of infections between groups in which infections from *Group 1* flow to *Group 2* at a rate ϕ, and infections from *Group 2* flow to *Group 3* at a rate τ. *Group 4* is infected from *Group 2* at a rate γ and from *Group 3* at a rate α. We assumed

that a non-pharmaceutical intervention is implemented at t_{interv} and generates a reduction ε in the hazard rate of infection. Figure S1 illustrates the schematic diagram of the model.

Vaccination was incorporated into the model through a distinct and separate COVID-19 natural history for the proportion of individuals undergoing the intervention.

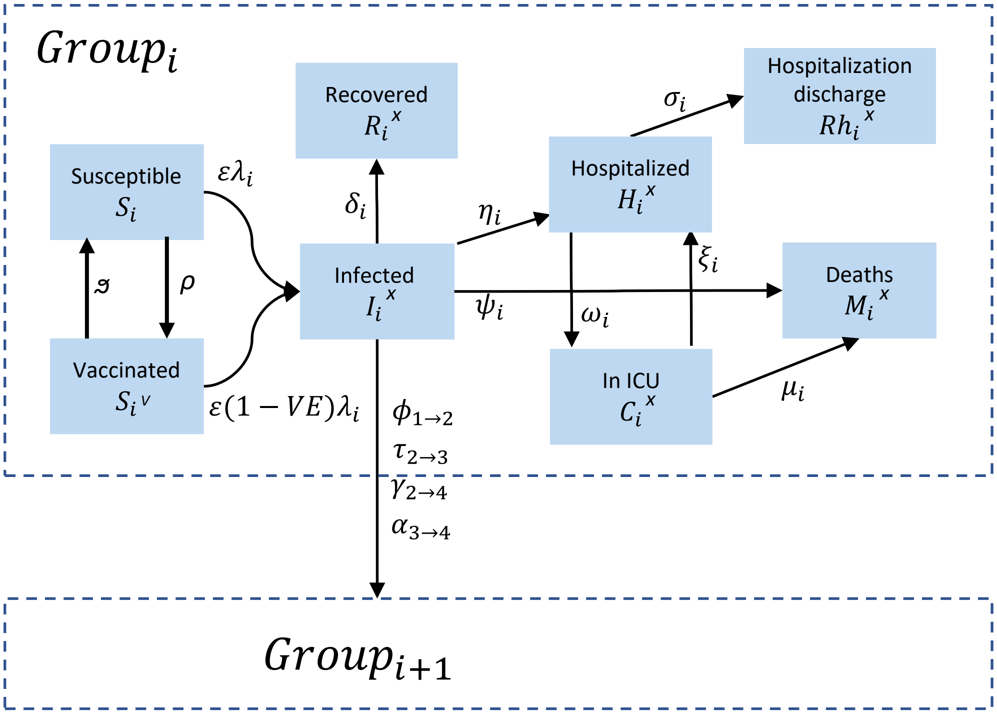

Figure S1. Schematic diagram for the Susceptible-Infected-Hospitalized-Recovered-Dead (SIHRD) COVID-19 deterministic compartmental model.

No intervention branch of the equations

Counties with airports (Group 1):

$$\frac{dS_1}{dt} = \vartheta S_1^V - \lambda_1 S_1 \frac{I_1 + I_1^V}{N_1} - \rho_1 S_1$$

$$\frac{dI_1}{dt} = \lambda_1 S_1 \frac{I_1 + I_1^V}{N_1} - I_1(\delta + \eta_1 + \psi)$$

$$\frac{dH_1}{dt} = \eta_1 I_1 + \xi C_1 - H_1(\omega_1 + \sigma)$$

$$\frac{dC_1}{dt} = \omega_1 H_1 - C_1(\mu_1 + \xi)$$

$$\frac{dM_1}{dt} = \mu_1 C_1 + \psi I_1$$

$$\frac{dR_1}{dt} = \delta I_1$$

$$\frac{dRh_1}{dt} = \sigma H_1$$

Counties surrounding counties with airports (Group 2):

$$\frac{dS_2}{dt} = \vartheta S_2^V - S_2\left(\phi \frac{I_1 + I_1^V}{N_1} + \lambda_2 \frac{I_2 + I_2^V}{N_2}\right) - \rho_2 S_2$$

$$\frac{dI_2}{dt} = S_2(\phi \frac{I_1 + I_1^V}{N_1} + \lambda_2 \frac{I_2 + I_2^V}{N_2}) - I_2(\delta + \eta_2 + \psi)$$

$$\frac{dH_2}{dt} = \eta_2 I_2 + \xi C_2 - H_2(\omega_2 + \sigma)$$

$$\frac{dC_2}{dt} = \omega_2 H_2 - C_2(\mu_2 + \xi)$$

$$\frac{dM_2}{dt} = \mu_2 C_2 + \psi I_2$$

$$\frac{dR_2}{dt} = \delta I_2$$

$$\frac{dRh_2}{dt} = \sigma H_2$$

Counties with main highways (Group 3):

$$\frac{dS_3}{dt} = \vartheta S_3^V - S_3\left(\tau\frac{I_2 + I_2^V}{N_2} + \lambda_3\frac{I_3 + I_3^V}{N_3}\right) - \rho_3 S_3$$

$$\frac{dI_3}{dt} = S_3\left(\tau\frac{I_2 + I_2^V}{N_2} + \lambda_3\frac{I_3 + I_3^V}{N_3}\right) - I_3(\delta + \eta_3 + \psi)$$

$$\frac{dH_3}{dt} = \eta_3 I_3 + \xi C_3 - H_3(\omega_3 + \sigma)$$

$$\frac{dC_3}{dt} = \omega_3 H_3 - C_3(\mu_3 + \xi)$$

$$\frac{dM_3}{dt} = \mu_3 C_3 + \psi I_3$$

$$\frac{dR_3}{dt} = \delta I_3$$

$$\frac{dRh_3}{dt} = \sigma H_3$$

<u>Low risk counties not surrounding counties with airports and without main highways (Group 4)</u>:

$$\frac{dS_4}{dt} = \vartheta S_4^V - S_4\left(\gamma\frac{I_2 + I_2^V}{N_2} + \alpha\frac{I_3 + I_3^V}{N_3} + \lambda_4\frac{I_4 + I_4^V}{N_4}\right) - \rho_4 S_4$$

$$\frac{dI_4}{dt} = S_4\left(\gamma\frac{I_2 + I_2^V}{N_2} + \alpha\frac{I_3 + I_3^V}{N_3} + \lambda_4\frac{I_4 + I_4^V}{N_4}\right) - I_4(\delta + \eta_4 + \psi)$$

$$\frac{dH_4}{dt} = \eta_4 I_4 + \xi C_4 - H_4(\omega_4 + \sigma)$$

$$\frac{dC_4}{dt} = \omega_4 H_4 - C_4(\mu_4 + \xi)$$

$$\frac{dM_4}{dt} = \mu_4 C_4 + \psi I_4$$

$$\frac{dR_4}{dt} = \delta I_4$$

$$\frac{dRh_4}{dt} = \sigma H_4$$

Intervention branch of the equations

Counties with airports (Group 1):

$$\frac{dS_1^V}{dt} = \rho_1 S_1 - \lambda_1 (1\text{-}VE_s) S_1^V \frac{I_1 + I_1^V}{N_1} - \vartheta S_1^V$$

$$\frac{dI_1^V}{dt} = \lambda_1 (1\text{-}VE_s) S_1^V \frac{I_1 + I_1^V}{N_1} - I_1^V (\delta + \eta_1 + \psi)$$

$$\frac{dH_1^V}{dt} = \eta_1 I_1^V + \xi C_1^V - H_1^V (\omega_1 + \sigma)$$

$$\frac{dC_1^V}{dt} = \omega_1 H_1^V - C_1^V (\mu_1 + \xi)$$

$$\frac{dM_1^V}{dt} = \mu_1 C_1^V + \psi I_1^V$$

$$\frac{dR_1^V}{dt} = \delta I_1^V$$

$$\frac{dRh_1^V}{dt} = \sigma H_1^V$$

Counties surrounding counties with airports (Group 2):

$$\frac{dS_2^V}{dt} = \rho_2 S_2 - S_2^V \left(\phi (1\text{-}VE_s) \frac{I_1 + I_1^V}{N_1} + \lambda_2 (1\text{-}VE_s) \frac{I_2 + I_2^V}{N_2} \right) - \vartheta S_2^V$$

$$\frac{dI_2^V}{dt} = S_2^V (\phi (1\text{-}VE_s) \frac{I_1 + I_1^V}{N_1} + \lambda_2 (1\text{-}VE_s) \frac{I_2 + I_2^V}{N_2}) - I_2^V (\delta + \eta_2 + \psi)$$

$$\frac{dH_2^V}{dt} = \eta_2 I_2^V + \xi C_2^V - H_2^V (\omega_2 + \sigma)$$

$$\frac{dC_2^V}{dt} = \omega_2 H_2^V - C_2^V (\mu_2 + \xi)$$

$$\frac{dM_2^V}{dt} = \mu_2 C_2^V + \psi I_2^V$$

$$\frac{dR_2^V}{dt} = \delta I_2^V$$

$$\frac{dRh_2^V}{dt} = \sigma H_2^V$$

Counties with main highways (Group 3):

$$\frac{dS_3^V}{dt} = \rho_3 S_3 - S_3^V \left(\tau(1\text{-}VE_s)\frac{I_2 + I_2^V}{N_2} + \lambda_3(1\text{-}VE_s)\frac{I_3 + I_3^V}{N_3} \right) - \vartheta S_3^V$$

$$\frac{dI_3^V}{dt} = S_3^V \left(\tau(1\text{-}VE_s)\frac{I_2 + I_2^V}{N_2} + \lambda_3(1\text{-}VE_s)\frac{I_3 + I_3^V}{N_3} \right) - I_3^V(\delta + \eta_3 + \psi)$$

$$\frac{dH_3^V}{dt} = \eta_3 I_3^V + \xi C_3^V - H_3^V(\omega_3 + \sigma)$$

$$\frac{dC_3^V}{dt} = \omega_3 H_3^V - C_3^V(\mu_3 + \xi)$$

$$\frac{dM_3^V}{dt} = \mu_3 C_3^V + \psi I_3^V$$

$$\frac{dR_3^V}{dt} = \delta I_3^V$$

$$\frac{dRh_3^V}{dt} = \sigma H_3^V$$

Low risk counties not surrounding counties with airports and without main highways (Group 4):

$$\frac{dS_4^V}{dt} = \rho_4 S_4 - S_4^V \left(\gamma(1\text{-}VE_s)\frac{I_2 + I_2^V}{N_2} + \alpha(1\text{-}VE_s)\frac{I_3 + I_3^V}{N_3} + \lambda_4(1\text{-}VE_s)\frac{I_4 + I_4^V}{N_4} \right) - \vartheta S_4^V$$

$$\frac{dI_4^V}{dt} = S_4^V \left(\gamma(1\text{-}VE_s)\frac{I_2 + I_2^V}{N_2} + \alpha(1\text{-}VE_s)\frac{I_3 + I_3^V}{N_3} + \lambda_4(1\text{-}VE_s)\frac{I_4 + I_4^V}{N_4} \right) - I_4^V(\delta + \eta_4 + \psi)$$

$$\frac{dH_4^V}{dt} = \eta_4 I_4^V + \xi C_4^V - H_4^V(\omega_4 + \sigma)$$

$$\frac{dC_4^V}{dt} = \omega_4 H_4^V - C_4^V(\mu_4 + \xi)$$

$$\frac{dM_4^V}{dt} = \mu_4 C_4^V + \psi I_4^V$$

$$\frac{dR_4^V}{dt} = \delta I_4^V$$

$$\frac{dRh_4^V}{dt} = \sigma H_4^V$$

Given the evidence for declining COVID-19 incidence due to many factors such as social distancing and temporal lockdown, hospitalizations and mortality due to better treatment, the temporal variation of $\varepsilon_y(t)$, were *y* = contact rate, hospitalizations, or mortality, was characterized by a Wood-Saxon function (Velicia 1987;Woods et al. 1954):

$$\varepsilon_y(t) = \varepsilon_0 \left(1 + \frac{Z_y}{1 + \exp\left[\left(t - T_y \right) / D_y \right]} \right)$$

Here, ε_0 is the asymptotic value that describes the contact rate well after the transition, Z_y is the level of change in $\varepsilon_y(t)$ during the transition from $\varepsilon_0\left(1+Z_y\right)$ before the transition to ε_0 after the transition, D_y describes the transition duration parameter, and T_y is the turning point year at which the contact rate crosses half way towards its asymptotic value of ε_0.

These parameters were obtained by fitting the model to available country-specific incidence data.

Table S1. Definition of population variables

Symbol	Definition
S_i^X	Susceptible population in group *i*, where index *X* marks the intervention status
I_i^X	Infected population in group *i*, where index *X* marks the intervention status
H_i^X	Hospitalized population in group *i*, where index *X* marks the intervention status
C_i^X	Population admitted into ICU in group *i*, where index *X* marks the intervention status
R_i^X	Recovered population in group *i*, where index *X* marks the intervention status
Rh_i^X	Population discharged from hospitals in group *i*, where index *X* marks the intervention status
M_i^X	Deaths in group *i*, where index *X* marks the intervention status
N_i	Total population in group *i*
λ_i	Hazard rate of infection for spatial risk group *i*
δ	Natural recovery rate without hospitalization
η_i	Hospitalization rate for spatial risk group *i*
ω_i	Admission rate of ICU admission rate in spatial risk group *i*
ξ	Recovery rate from ICU
σ	Discharge rate from hospital
μ_i	Mortality rate for individuals in ICU in spatial risk group *i*
ψ	Mortality rate for infected individuals
ϕ	Rate of infection flow from group 1 to group 2
τ	Rate of infection flow from group 2 to group 3

γ	Rate of infection flow from group 2 to group 4
α	Rate of infection flow from group 3 to group 4
ρ	Vaccination rate
ϑ	Waning rate of vaccine immunity (i.e., duration of vaccine protection)
VE_S	Fractional reduction in the hazard rate of infection

Table S2. Definition of parameters

Symbol	Definition	Estimated value	Justification
λ_i; i= 1, 2, 3, 4	Hazard rate of infection for spatial risk group *i*	1= 0.28<br>2= 0.25<br>3= 0.08<br>4= 0.12	Fitted parameter
ε_c	Wood-Saxon time dependent contact rate	T_c= 20.02<br>D_c=18.57<br>Z_c= 2.19	Fitted parameter
$1/\delta$	Natural recovery rate without hospitalization	4.01 days	Fitted parameter
η_i; i= 1, 2, 3, 4	Hospitalization rate for spatial risk group *i*	1= 0.014<br>2= 0.011<br>3= 0.005<br>4= 0.002	Fitted parameter
ε_h	Wood-Saxon time dependent hospitalization rate	T_h= 33.26<br>D_h=12.91<br>Z_h= 0.10	Fitted parameter
ω_i; i= 1, 2, 3, 4	Admission rate of ICU admission rate in spatial risk group *i*	1= 0.004<br>2= 0.019<br>3= 0.006<br>4= 0.009	Fitted parameter
ξ	Recovery rate from ICU	0.14	Fitted parameter
σ	Discharge rate from hospital	0.51	Fitted parameter
μ_i; i= 1, 2, 3, 4	Mortality rate for individuals in ICU in spatial risk group *i*	1= 0.092<br>2= 0.013<br>3= 0.015<br>4= 0.091	Fitted parameter
ε_m	Wood-Saxon time dependent mortality rate	T_m= 30.42<br>D_m=18.57<br>Z_m= 2.19	Fitted parameter
ψ	Mortality rate for infected individuals	0.0048	Fitted parameter
ϕ	Rate of infection flow from group 1 to group 2	0.021	Fitted parameter
τ	Rate of infection flow from group 2 to group 3	0.071	Fitted parameter

γ	Rate of infection flow from group 3 to group	0.068	Fitted parameter
α	Rate of infection flow from group 2 to group 4	0.009	Fitted parameter

Table S3. Impact of different vaccination scenarios on COVID-19 related infections and hospitalizations. The effect was defined in averted cases, and the number of vaccinations needed per case/hospitalization averted (i.e., population-level effectiveness of a vaccination program). Scenario 1, one million vaccine doses were distribut-ed homogeneously across Ohio's different spatial risk areas. Scenario 2, 60% of all vaccines were administered to Group 3 and Group 4 (i.e., identified as areas with low infection intensity), while 40% of the vaccines were equally distributed across Group 1 and Group 2 (i.e., identified as areas with high infection intensity). Scenario 3, 90% were equally distributed across Group 1 and Group 2, and only 10% of all vaccines were allocated to Group 3 and Group 4. Credible intervals (Crls)

**Scenario**	**Total number of infections averted (95% Crls)**	**Vaccines per Infection averted (95%Crls)**	**Total number of Hospitalizations averted (95% Crls)**	**Vaccines per hospitalization averted (95%Crls)**
Scenario 1	3024 (2835–3054)	3.91 (3.85-4.17)	168 (152-169)	71 (69-77)
Scenario 2	2149 (1997-2177)	5.17 (4.18-5.5)	116 (104-117)	96 (94-105)
Scenario 3	3756 (3528-3786)	3.15 (3.10-3.40)	213 (193-214)	56 (55-60)

ADDITIONAL FIGURES

Figure S2. Model fitting to cumulative number of COVID-19 cases in Ohio**.**

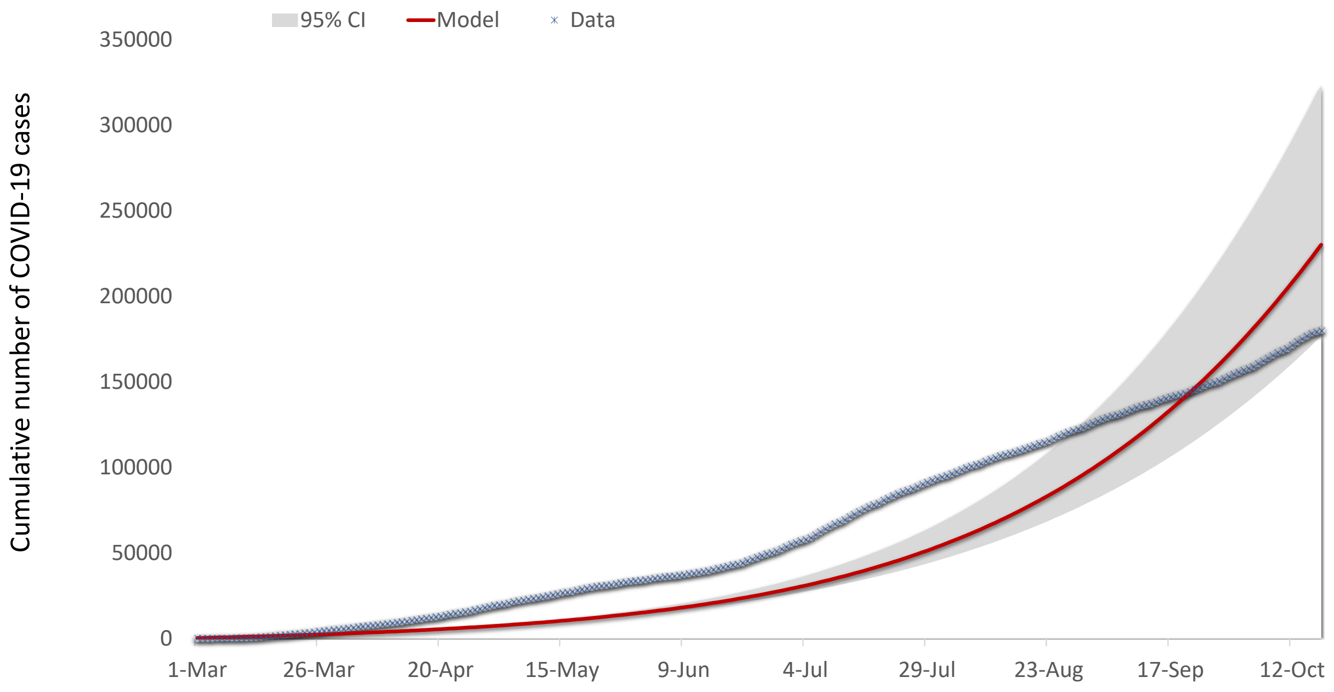

Figure S3. Cumulative number of hospitalizations under different vaccination scenario.

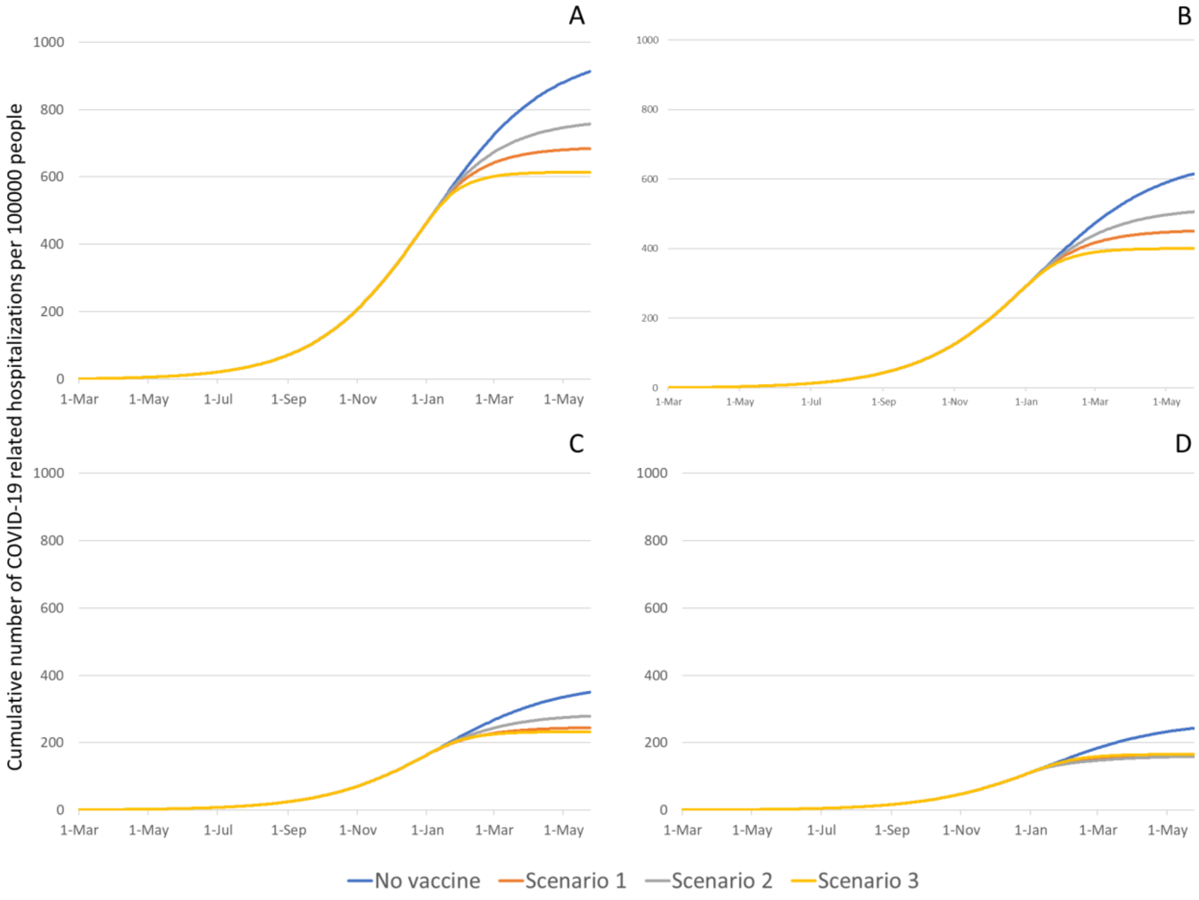

Figure S4. **Sensitivity analysis.** Different vaccine efficacies were assumed for the rural areas (efficacy of 50%) compared to urban areas (efficacy of 90%),

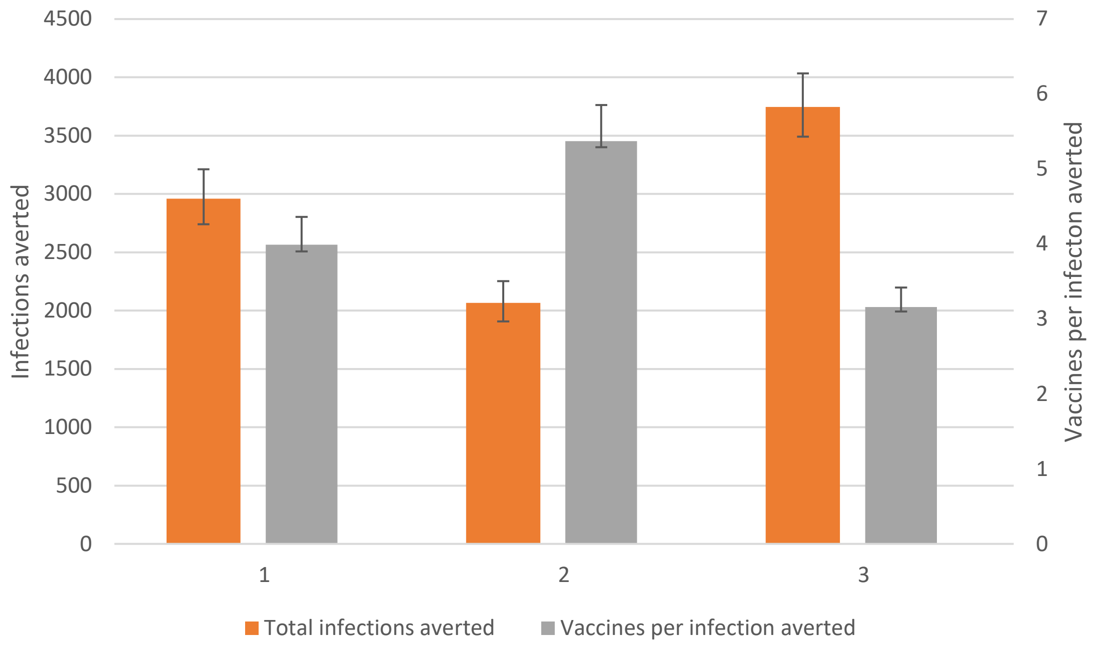

Supplement: Supplementary file 1 [file vaccines-09-01242-s001.zip › vaccines-1400003-supplementary.pdf]
